# Joining Forces for Cancer Treatment: From “TCR versus CAR” to “TCR and CAR”

**DOI:** 10.3390/ijms232314563

**Published:** 2022-11-23

**Authors:** Karin Teppert, Xueting Wang, Kathleen Anders, César Evaristo, Dominik Lock, Annette Künkele

**Affiliations:** 1 Miltenyi Biotec B.V. & Co. KG, 51429 Bergisch Gladbach, Germany; 2 German Cancer Consortium (DKTK), 10117 Berlin, Germany; 3 German Cancer Research Center (DKFZ), 69120 Heidelberg, Germany; 4 Department of Pediatric Oncology and Hematology, Charité-Universitätsmedizin Berlin, Corporate Member of Freie Universität Berlin, Humboldt-Universität zu Berlin, and Berlin Institute of Health, 13353 Berlin, Germany

**Keywords:** immunotherapy, tumor immunology, adoptive T cell therapies, immune synapse, signaling, endosomal trafficking, T cell engineering, chimeric antigen receptor, T cell receptor

## Abstract

T cell-based immunotherapy has demonstrated great therapeutic potential in recent decades, on the one hand, by using tumor-infiltrating lymphocytes (TILs) and, on the other hand, by engineering T cells to obtain anti-tumor specificities through the introduction of either engineered T cell receptors (TCRs) or chimeric antigen receptors (CARs). Given the distinct design of both receptors and the type of antigen that is encountered, the requirements for proper antigen engagement and downstream signal transduction by TCRs and CARs differ. Synapse formation and signal transduction of CAR T cells, despite further refinement of CAR T cell designs, still do not fully recapitulate that of TCR T cells and might limit CAR T cell persistence and functionality. Thus, deep knowledge about the molecular differences in CAR and TCR T cell signaling would greatly advance the further optimization of CAR designs and elucidate under which circumstances a combination of both receptors would improve the functionality of T cells for cancer treatment. Herein, we provide a comprehensive review about similarities and differences by directly comparing the architecture, synapse formation and signaling of TCRs and CARs, highlighting the knowns and unknowns. In the second part of the review, we discuss the current status of combining CAR and TCR technologies, encouraging a change in perspective from “TCR versus CAR” to “TCR and CAR”.

## 1. Introduction

**Figure 1:**
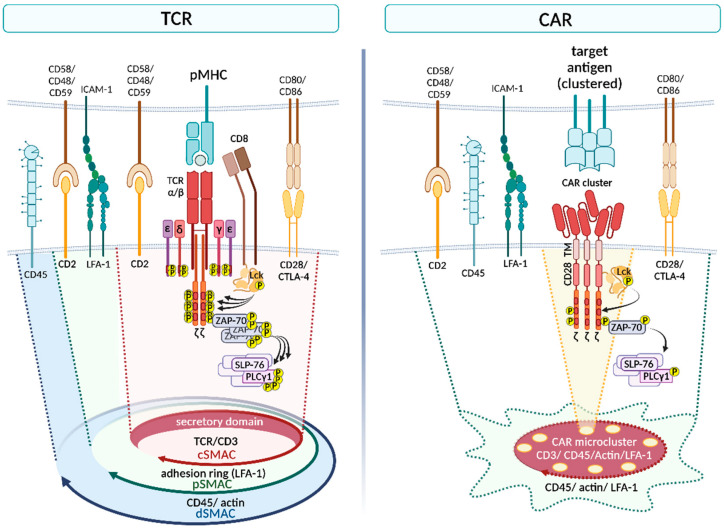
Interface of TCR and CAR, illustrating differences in immunological synapse formation and essential signaling elements (created with BioRender.com).

The beginning of adoptive T cell-based immunotherapies goes back to the 1980s when Rosenberg and colleagues successfully demonstrated the anti-cancer potential of tumor-infiltrating lymphocytes (TILs) [1]. Although TILs have shown encouraging results in several clinical trials [2,3,4,5], difficulties were reported in regard to the isolation and manufacturing of tumor-specific TILs [6]. This led to the approach of engineering T cells to express defined tumor-specific receptors, which are generally classified into T cell receptors (TCRs) and chimeric antigen receptors (CARs) [7]. CAR T cell therapy has led to great success in the treatment of hematological malignancies, resulting in the approval of several CAR products by the US Food and Drug Administration (FDA) [8]. However, antigen escape, therapy-associated toxicities and poor efficacy in solid tumors are some of the currently faced challenges in CAR T cell immunotherapy [9]. While transgenic TCRs have shown encouraging results in the treatment of solid tumors [10,11,12,13,14,15,16], the restriction to human leukocyte antigens (HLAs) constitutes a major constraint [17]. In this review, we outline the similarities and differences between TCRs and CARs in regard to architecture, immunological synapse formation and signaling, in order to establish a better understanding of the thereof resulting advantages and limitations. The main differences are summarized in Table 1 and illustrated in Figure 1.

## 2. Part I—TCR versus CAR

### 2.1. TCR versus CAR: Structure

T cells recognize intracellularly processed peptides [38,39,40] that are presented on major histocompatibility complexes (pMHCs) [41] through their endogenous T cell receptor (TCR) [42]. The majority of TCRs in humans are characterized by their heterodimeric architecture, assembled by an alpha and a beta chain (αβTCR), both consisting of a constant and a variable region. A minority of T cells express TCRs composed of a gamma and delta chain (γδTCR) which are not further addressed in this review. The variable region of an αβTCR (in the following only TCR) contains the three loop-forming complementary-determining regions CDR1, CDR2 and CDR3, being the main determinant for binding specificity [43]. Those structurally hypervariable CDRs are flanked by framework regions, which were reported to support CDR conformation. T cells are generally subdivided into CD8^+^ and CD4^+^ T cells and bind with their TCR to antigens displayed on class I and class II MHC molecules, respectively [41,44]. In humans, MHC class I and class II are referred to as HLA class I and HLA class II and are further subdivided into different HLA allele groups: HLA-A/-B/-C and HLA-DR/-DQ/-DM/-DP, respectively [45]. Hence, ligand recognition through the TCR is therefore restricted to both a specific peptide sequence and the peptide-presenting HLA molecule [46].

The extracellular domain of a CAR consists most often of an antibody-derived single-chain variable fragment (scFv) [47] but can also be composed of a natural ligand or receptor domain [48]. Similar to TCRs, the scFv variable heavy and light chains both also contain three CDRs flanked by framework residues. Despite the general similarities in terms of architecture and formation through genetic rearrangement of the variable portion of both scFv-based CARs and TCRs [46,49], more detailed studies pointed out that compared to antibodies, TCRs display longer CDR3 loops [50], a higher number of negatively charged amino acids in CDR1 and CDR2 [51] and structurally more variable loops [49]. Besides this, conventional CARs do not target processed proteins presented via MHC molecules but instead bind to unprocessed antigens expressed on the cell surface, including proteins, glycolipids and carbohydrates. With the goal to broaden the range of CAR targets by also including intracellular proteins, some work was also focused on generating pMHC-directed CARs, so-called TCR-mimicking CARs [52,53,54,55,56]. Moreover, several approaches aimed at engineering TCRs attached to antibody-derived antigen-binding domains to achieve HLA independence [57,58,59]. This underlines the steady interchange of new learning between TCRs and CARs, allowing for the implementation of advantageous features at both ends. TCR signaling requires not only engagement with the pMHC but also, with rare exceptions, interaction with the co-receptor CD4 or CD8 [60,61,62] and always an intracellular association with the CD3 complex [63], consisting of the three dimers CD3εδ, CD3εγ and CD3ζζ [64,65]. The cytoplasmic tails of CD3ε,γ,δ and CD3ζ contain one and three immunoreceptor tyrosine-based activation motifs (ITAMs), respectively [26]. ITAM phosphorylation upon TCR engagement with pMHC leads to intracellular signal amplification (more detailed in Section 2.3.1 Proximal Signaling) [26]. Except for CARs harboring a CD3ζ transmembrane domain [66], CARs are usually unable to associate with the endogenous CD3 complex and are therefore equipped with a single CD3ζ signaling domain in their intracellular part. This means that a conventional CAR only displays 3 ITAMs, whereas the TCR/CD3 complex contains a total of 10 ITAMs [26]. It has been postulated that increasing the number of ITAMs can lead to higher sensitivity and potency, thereby minimizing the required number of engaged receptors for an equivalent response [26,67]. However, in vivo studies demonstrated that CD19 CARs harboring a mutated CD3ζ signaling domain with only one functional ITAM located proximally to the membrane and ablation of the second and third ITAMs (1XX) outperformed the standard CARs containing a full CD3ζ chain, while inactivation of the two N-terminal ITAMs (XX3) demonstrated decreased efficiency [68,69]. Despite those advances regarding optimal ITAM positioning and number in the CD3ζ chain, the diverse roles of the other CD3 chains are currently extensively studied, not only in regard to the TCR but also as additional or alternative domains in the CAR [70]. Additional incorporation of the CD3ε chain was, for instance, reported to allow for a more balanced CAR response with enhanced persistence and reduced cytokine secretion, tonic signaling and exhaustion [70].

The first generation of CARs is characterized by an extracellular scFv, linked to a transmembrane domain via a spacer region, followed by the intracellular CD3ζ signaling domain [71]. This build-up was insufficient to produce potent and persistent T cell responses and was further optimized and step-wise extended through additional domains to increase in vivo efficacy. Integration of a co-stimulatory domain derived, e.g., from CD28 or 4-1BB led to the development of second-generation CARs (here referred to as 28ζ and BBζ CAR, respectively) with the ability to produce IL-2 and remain functional upon repeated antigen encounter [72]. Second-generation CARs were the first format approved by the FDA in 2017 [73,74]. Third-generation CARs are characterized by a combination of two co-stimulatory domains, while fourth- and fifth-generation CARs additionally incorporate a transgene for pro-inflammatory cytokine production or a JAK-STAT3/5 pathway-activating domain, respectively [75,76,77]. The idea of providing a CD3ζ domain, a co-stimulatory domain and also cytokines was adopted from natural T cell signaling, which requires three signals for full activation and effector function: first, stable pMHC binding, second, co-stimulation and, third, signaling through cytokine receptors [78].

Up to now, each of the domains in the previously described second-generation CAR has been exchanged or modified, displaying certain characteristics and different effects on functionality [79]. However, one optimal composition or the “magic bullet”, which would be suitable in all settings, has not yet been found [80]. Despite modifications of the CAR endodomains, it was shown that CAR functionality is strongly affected by the length and type of the spacer, linking the transmembrane domain and scFv [81]. Depending on scFv affinity, target epitope location or distance to the cell surface, it was shown that linker length influences CAR T cell activation, tonic signaling, phenotype, migration and overall potency [82,83,84,85]. This leads to the conclusion that every CAR molecule needs to be constructed individually and all of the CAR domains should consequently be adjusted to one another while considering tumor cell type as well as the target antigen size and density [81,82,86,87].

### 2.2. TCR versus CAR: Activation upon Stimulation

#### 2.2.1. Antigen Engagement for Initiation of Activation

T cell signaling through TCRs is initiated upon recognition of antigenic peptides presented on MHC molecules and is further strengthened through the binding of co-receptors CD4 or CD8 to MHC [88,89]. In contrast, CAR signaling is induced after the engagement of unprocessed antigens in an MHC-independent manner [47]. However, how the antigen recognition by TCRs and CARs is transmitted into intracellular signaling remains incompletely understood. There are three main theories describing signaling mechanisms triggered by the TCR: (1) nanoclustering, (2) kinetic segregation model and (3) mechanosensing. First, despite the fact that a single TCR complex can act as a predominant receptor driving ligand recognition [90], it has been observed that the nanoclustering of TCR is a process of antigen discrimination [91]. This is regulated by the differentiation state of T cells, as memory T cells, which have increased antigen sensitivity compared to naïve T cells and tend to form dense signaling-competent TCR clusters [92]. Second, the kinetic segregation model describes a size-dependent protein segregation mechanism. After T cells encounter a high-affinity cognate antigen, molecules containing shorter ectodomains such as TCRs and co-receptors can still diffuse to the “close-contact zone” at the interface between the T cell and antigen-presenting cell (APC), whereas molecules with longer ectodomains, such as CD45 tyrosine phosphatase, are excluded from the interface. This leads to a change in the kinase:phosphatase balance in the proximity of the TCR complex, thereby initiating TCR phosphorylation [93,94,95]. Third, increasing evidence indicates that the TCR acts as an anisotropic mechanosensor, which can discriminate antigens in a force-dependent manner. TCR:pMHC binding exerts force leading to structural transitions of the TCR complex and local cytoskeleton rearrangement, eventually leading to downstream signaling [96,97,98,99]. These three theories have shed light on different aspects and are not necessarily mutually exclusive. Instead, they could all contribute to kinetic proof-reading, a process of signaling accumulation to discriminate self from foreign ligands prior to dissociation of the TCR:pMHC complex [100]. Compared to TCRs, knowledge about signaling initiation upon CARs still remains to be elucidated. There is evidence suggesting that receptor clustering is an important step to initiate signaling [101] and supporting that the proof-reading kinetics of CARs resemble those of TCRs [102].

The sensitivity of TCRs was described to be up to 100-fold higher than that of CARs [25,103]. Although TCRs are generally characterized by low-affinity binding, it was shown that already a single pMHC complex was enough to trigger TCR signaling [18,19]. In contrast, CAR signaling requires clustering, which consequently means that a higher and sufficient number of antigens needs to be expressed on the target cell surface [20,24,57,104]. Hence, CAR and TCR signaling both depend on the optimal balance between affinity and antigen density. The comparison of CARs and transgenic HLA-independent TCRs, generated to target the same surface antigen, demonstrated that the structure of the TCR and its association with the complete CD3 complex is indeed the key to the higher sensitivity [58,59,105]. More precisely, it is thought that several structural features, such as the number and positioning of ITAMs [26,67], the interaction with the co-receptor CD4 or CD8 [106] and the interaction with co-stimulatory and adhesion molecules such as CD2 and LFA-1 [27], account for the more efficient signal transduction in TCRs [28], resulting in higher sensitivity and a lowered activation threshold [28].

#### 2.2.2. Formation of the Immunological Synapse

The immunological synapse (IS) is the structure at the interface between the T cell and its target cell or the APC, and is formed within minutes of TCR:pMHC or CAR:antigen binding. Upon IS formation, molecular interactions, cytoskeletal rearrangement and dynamic regulation take place, and they are essential for subsequent cell activation.

Typically, the TCR-IS is characterized as a well-organized structure. Kupfer and colleagues named this radially symmetric compartment the supramolecular activation cluster (SMAC), and it consists of three parts. The central part (cSMAC) includes TCR-MHC, intracellular signaling molecules and co-stimulatory (CD28, ICOS, 4-1BB, etc.)/co-inhibitory (PD-1/TIM-3/LAG-3, etc.) interaction clusters. The peripheral ring (pSMAC) surrounds the cSMAC and is formed by adhesion molecules. Finally, the distal ring (dSMAC) contains molecules with larger ectodomains such as CD45 [33]. This “bull’s eye” pattern has been observed in certain T cell subsets including cytotoxic T lymphocytes (CTLs) [107,108], naïve CD4 [109], Th1 cells and when contacting B cells, tumor cells and the artificial planar lipid bilayer. However, “non-classical ISs”, as well as motile kinapse (a transient and dynamic structure at the interface), have also been observed under various conditions [110]. For instance, “multifocal ISs”, characterized by adhesion molecules dispersed among multiple TCR:MHC accumulations, have been reported in Th2 cells [111,112] at the interface between T cells and dendritic cells [32] or upon contact between immature double-positive thymocytes and the artificial lipid bilayer [113]. Thus, the formation of the bull’s eye structure with well-defined cSMACs and pSMACs is not a requirement for T cell activation. Initially, it was proposed that the proper accumulation of TCR-proximal molecules in the cSMAC is required to initiate signaling [114], yet the variations on SMACs indicate that this is not the case. Moreover, phosphorylated signaling proteins such as lymphocyte-specific protein tyrosine kinase (Lck) and Zeta-chain-associated protein kinase 70 (ZAP-70) are found prior to the formation of mature ISs, and the majority of phosphorylated signaling proteins are localized in the pSMAC [33,115]. Using an artificial planar lipid bilayer has demonstrated that small TCR microclusters were firstly found at the periphery of the interface, which was associated with signaling molecules including Lck, ZAP-70, the linker for activation of T cells (LAT) and SH2 domain containing leukocyte protein of 76kDa (SLP-76). Meanwhile, tyrosine phosphorylation and calcium signaling also take place in pSMAC. However, as TCR microclusters migrate toward the cSMAC, TCR-proximal signaling molecules including CD28 and protein kinase C-θ dissociate from the microclusters [116,117], indicating that TCR signaling is initiated and sustained in the pSMAC. In addition, rearrangement of the actin cytoskeleton also occurs during IS formation [118,119], which is required for the centripetal movement of microclusters, as interfering with actin rearrangement through myosin IIa inhibition led to diminished proximal TCR signaling including the phosphorylation of ZAP-70 and LAT, indicating that actin polymerization is essential for proximal signaling [120]. Thus, cSMAC may rather play a role in the downregulation of signaling. Cemerski and colleagues’ study revealed that, depending on the stimulation strength, both signaling initiation and downregulation can occur in cSMAC [121]. Upon stimulation of weak agonists, signaling from cSMAC can be detected shortly after signaling events from pSMAC occur [121]. Indeed, accumulating evidence supports the idea that cSMAC plays a dual role in both sustaining and terminating the TCR-dependent signaling [119,122]. Cbl-b, a ubiquitin ligase and strong ubiquitin signal were found to concentrate in the cSMAC, supporting that cSMAC is associated with the internalization and degradation of the TCR complex [123]. Moreover, cSMAC formation is important for the cytolytic function of CTLs, meaning site-directed secretion of cytolytic granules to target the membrane through the polarization of the microtubule cytoskeleton [108,124].

It has been proposed that the quality of CAR-IS can predict CAR T cell efficiency [125]. However, in contrast with TCR-IS, CAR-IS is not well studied along with a lot of open questions needed to be addressed. Davenport and colleagues performed a cell-based side-by-side comparison of IS formation between HER2-28ζ CAR and OTI-TCR on CD8^+^ CTLs [29]. They observed a disorganized CAR-IS structure with a multifocal pattern, where elements of cSMAC and pSMAC are not separated but rather merged together, and no ring structure of adhesion molecules surrounding CAR clusters was detected in CAR-IS. This might be due to the fact that the ectodomain is larger in CARs than in TCRs [126]. In addition, both proximal (Lck and ZAP-70) and distal (ERK, cytotoxic granule delivery) signaling was induced and decreased faster in CAR-IS than TCR-IS, and the CAR-IS formation was less dependent on LFA-1:ICAM-1 interaction as compared to the TCR. The non-classical CAR-IS structure was further confirmed using an artificial planar lipid bilayer and a third-generation CAR (CD19-28-BBζ) [21], where segregation of CD45 from CAR clusters was observed. Moreover, they showed that LAT, an essential scaffold protein for TCR signaling, is not necessarily required for the downstream SLP-76 phosphorylation. In addition, the overall binding strength, or the avidity between the CAR and its target, is a critical factor determining CAR T cell response. Differential requirement of avidity has been demonstrated by comparing a liquid tumor (CD19+ or BCMA+) and glioblastoma. Unlike a liquid tumor, engagement of the IFNγ receptor was required for treating a solid tumor [127], indicating that a certain avidity threshold needs to be reached for sufficient CAR T cell response. However, it is still not clear whether these findings are unbiased or based on certain bias introduced by model selection and assay design. Indeed, further investigation is needed to elucidate how other molecules such as co-stimulatory/co-inhibitory elements are involved in CAR-IS formation or stabilization, whether different IS structures are formed in certain T cell subsets and how the cytoskeleton is regulated and its consequence. As the cSMAC of TCR-IS plays a dual role in both sustaining and terminating signaling, it is important to study the impact of cSMAC absence in CAR-IS.

### 2.3. TCR versus CAR: Signaling Cascade

Signaling events are triggered upon antigen engagement and during immunological synapse/kinapse formation. The subsequent signaling cascades then lead to the activation of T cells. The utilization of TCR intracellular pathways to drive T cell activation is the fundament of CAR design. As the TCR/CD3 complex and the conventional CARs both have the CD3ζ intracellular domain, it is expected that some signaling cascades are shared for T cell activation and to induce a desired anti-tumor response. However, differences in signaling prevail due to the artificial constitution of CARs [23,128].

#### 2.3.1. Proximal Signaling

TCR signaling is largely initiated by a set of protein tyrosine kinases (PTKs) including the Src family tyrosine kinases Lck and Fyn. Lck is basally active [129], and it exists in soluble, membrane-anchored and coreceptor-bound forms [130,131]. After antigen engagement, Lck is recruited to the TCR complex and phosphorylates the ITAMs of CD3 subunits [129,132]. ITAM phosphorylation leads to the recruitment of ZAP-70 and subsequently phosphorylation and activation of ZAP-70. Once phosphorylated, ZAP-70 is able to initiate a series of phosphorylation cascades resulting in the assembly and activation of signaling complexes, which are important for propagating the TCR/PTK signal into late signaling outcomes. Two adaptor proteins are the most important targets of phosphorylated ZAP-70: the LAT and SLP-76 [133]. When phosphorylated by ZAP-70, LAT and SLP-76 can bind to specific signaling proteins through SH2 domains and form oligomeric signalosomes [134,135]. Multiple signaling proteins can be recruited to phosphorylated LAT, including phospholipase C gamma (PLCγ), the adapter growth factor receptor-bound protein 2 (GRB2) and GRB2-related adapter downstream of Shc (Gads). In addition, SLP-76 is indirectly associated with phosphorylated LAT through Gads. Recruitment of PLCγ activates the calcium and Ras/MAPK pathway [136], whereas the multivalent interactions between LAT, Gads and SLP-76 enable the reversible assembly of a protein cluster or the LAT signalosome, which enhances actin polymerization [137,138,139] and triggers Ras, Rac and Rho GTPas activation. In addition, TCR and CD28 co-stimulatory molecules activate the PI3K pathway, which is also associated with LAT and SLP-76 [140] and leads to calcium (Ca^2+^) influx and subsequent activation of the NFAT pathway.

Although signaling elements for proximal signaling of CARs and TCRs are similar, they differ in some aspects due to their artificial design in combining activating and co-stimulatory elements in one construct. Furthermore, there is a different requirement in antigen engagement, since TCRs have higher sensitivity to antigens than CARs, even though CARs have significantly higher affinity to antigens than TCRs [24], which has been shown to be associated with less efficient Lck recruitment and recycling to CARs than to TCRs [28]—probably due to the high redundancy of ITAMs in the TCR complex and less efficient CD4/CD8 coreceptor recruitment to CARs. Conventional CARs have the CD3ζ intracellular domain containing three ITAMs to transduce the signal for T cell activation. However, whether this finding can be translated to other systems remains to be elucidated. Upon the antigen binding of CARs, ZAP-70, SLP-76 and PLCγ are phosphorylated [126,141]. In contrast with TCRs, LAT might not be essential for CAR signaling as the loss of LAT had no impact on microcluster formation, actin remodeling and downstream signaling [21]. Depending on the CAR design, differences in kinetics and signaling magnitude have been observed. By comparing 28ζ and BBζ CARs targeting CD19 and ROR1, 28ζ CARs induced more rapid and intense phosphorylation of signaling intermediates and demonstrated a more effector-cell-like phenotype than BBζ CARs, which can be explained by the increased basal phosphorylation of the CD3ζ domain as well as greater Lck recruitment to 28ζ CARs [141]. Moreover, BBζ CARs have been shown to recruit the Themis-SHP1 phosphatase complex, which attenuates phosphorylation signaling [142].

#### 2.3.2. Downstream Signaling and Outcome

In principle, TCR and CAR downstream signaling is supposed to be similar, since the goal of introducing CARs is to redirect T cell specificity against the tumor and induce T cell effector function including proliferation, differentiation, and cytotoxicity through lytic granules and production of cytokines to achieve tumor clearance and sustained control [143]. However, optimal T cell activation requires not only signaling from the TCR receptor (signal 1) but also signaling from co-stimulatory molecules such as CD28 (signal 2) to prevent anergy, as well as soluble molecules such as cytokines to obtain full effector function (signal 3). Cell response is a consequence of signaling interplay across different pathways. Given the modular architecture of CARs and depending on the signaling domains integrated into the CAR, the signaling outcome can be remarkably different. It has been confirmed by different studies that changes in the signaling domain can lead to various CAR T cell responses with the mechanisms not fully understood to date. Here, we summarize the main downstream signaling pathways for T cell activation and modulation of the TCR and the corresponding knowledge about CARs.

#### 2.3.3. Calcium/NFAT Pathway

The second messenger Ca^2+^ plays an important role in T cell activation. Phosphorylated PLCγ cleaves phosphatidylinositol bisphosphate into inositol triphosphate (IP_3_) and diacylglycerol. IP_3_ then binds to the IP_3_/Ca^2+^ channel on the endoplasmic reticulum leading to Ca^2+^ release from the endoplasmic reticulum to the cytosol and promoting Ca^2+^ influx from extracellular through calcium-release-activated Ca^2+^ channels (CRAC) with a process termed store-operated Ca^2+^ entry (SOCE). The elevated Ca^2+^ can enable variable T cell effector functions in a magnitude- and duration-dependent manner [144]. Upon transient cytosolic Ca^2+^ increase, T cell motility, release of cytolytic granules by CTLs [145,146] and mitochondria translocations are induced [147]. Prolonged Ca^2+^ signaling can lead to the activation of the nuclear factor of activated T cells (NFAT), a key transcriptional regulator of the IL-2 gene and other cytokine genes, as well as subsequent cellular response including proliferation, cytokine production and differentiation [148,149,150,151].

A recent study on CD19-BBζ CAR T cells revealed the benefit of inhibiting calcium influx by using SOCE inhibitor BTP-2 both in vitro and in vivo. As calcium signaling was hyperactivated via sustained tonic signaling in these CAR T cells, treatment with BTP-2 rendered CAR T cells less exhausted and terminally differentiated with a metabolic profile of downregulated glycolysis [152].

#### 2.3.4. Ras/ERK/AP-1 and NF-κB Pathway

Diacylglycerol and cytosolic Ca^2+^ induce the activation of protein kinase C. Diacylglycerol can activate Ras guanyl-nucleotide-releasing proteins (RasGRPs) either directly through recruitment mechanisms or indirectly through PKC-mediated phosphorylation [153]. Subsequently, RasGRPs and SOS activate the MAPK/ERK pathway which then induces the activation of the transcription factor activator protein-1 (AP-1), a transcriptional complex formed by c-Jun and c-Fos, as well as B-cell lymphoma 2 (Bcl-2), which are involved in the cell cycle, cytokine production and cell apoptosis [154].

On the other hand, activated protein kinase C-ζ phosphorylates caspase recruitment domain-containing membrane-associated guanylate kinase protein-1 (CARMA1) leading to the recruitment of B cell lymphoma 10 (BCL10), mucosa-associated lymphoid tissue lymphoma translocation gene 1 (MALT1) and tumor necrosis TNF receptor associated factor 6 (TRAF6) and the formation of the CARMA1-BCL10-MALT1 complex [155,156]. This complex activates the IκB kinase (IKK) complex and leads to subsequent phosphorylation and ubiquitination of IκB and release of nuclear factor-κB (NF-κB), which regulates numerous genes critical for survival, proliferation, differentiation and cytokine production [157,158].

CARs with 4-1BB as a co-stimulatory domain have outperformed CD28 co-stimulated CARs in persistence and enrichment in a central memory-like state, indicating that distinct proliferation and survival signals are mediated by these two molecules [159,160]. Indeed, it has been shown only BBζ CARs activated noncanonical NF-κB (ncNF-κB) signaling after ligand engagement, and interfering with this pathway resulted in the reduced expansion and survival of CD19-BBζ T cells as well as accumulation of pro-apoptotic protein Bim [161], providing new possibility in manipulating CAR T cell responses.

#### 2.3.5. PI3K/AKT/mTOR Pathway

The phosphoinositide 3 kinase (PI3K)/Akt/mammalian (or mechanistic) target of the rapamycin (mTOR) pathway is a key regulator of cell proliferation. P85 is a subunit of PI3K, which can associate with both LAT and SLP-76 [162,163] and triggers activation of the PI3K pathway [140]. In addition, upon ligation of CD28 to B7.1 and B7.2 molecules, CD28 is phosphorylated by Src family tyrosine kinase, leading to the binding and activation of AKT by p85 and subsequent mTOR activation [164]. In addition, PI3K and Akt pathways are required for T cells to increase their glycolytic rate upon stimulation [165].

Studies on CAR signaling have demonstrated a more rapid and stronger early response of 28ζ CARs associated with PI3K signaling and signal transducer and activator of transcription 3 (STAT3) as compared to BBζ CARs [126,141], which might be due to differentiation of 28ζ CAR T cells to the short-lived, terminally differentiated effector state. In addition, in solid tumors, IL-2 secretion from 28ζ CAR T cells has been shown to promote Treg proliferation and thereby suppress the CAR T cell response. Modification on the Lck binding moiety in the intracellular part of CD28 demonstrated reduced IL-2 production and enhanced antitumor activity in the presence of Tregs [166]. 28ζ CAR bearing this modification outperformed BBζ CAR against prostate cancer [167], suggesting that excessive CD28-derived proximal signaling limited CAR T cell persistence, but adequate CD28 signaling could be a better option compared to 4-1BB in specific cases.

#### 2.3.6. Endosomal Trafficking and Lysosomal Degradation

Maintenance of a certain surface expression level of TCR complexes and CARs is critical for a sustained T cell response and is regulated by endocytosis, a process by which cells absorb external material by engulfing it with the cell membrane and is involved in the recycling and degradation of receptors. Coordinated by different Rab GTPases, several mechanisms of endocytosis have been characterized, which can be divided into two categories: clathrin-dependent endocytosis (CDE) and clathrin-independent endocytosis (CIE) [168]. The CDE pathway has been comprehensively analyzed with clathrin and its adaptor AP2 as major players [169]. Five CIE-independent pathways have been proposed including FEME (fast endophilin-mediated endocytosis), caveolae-associated endocytosis, CLIP/GEEC endocytosis and Arf6-mediated and flotillin-mediated endocytosis [34]. In contrast with CDE, processes involved in CIE remain to be fully elucidated. The internalized vesicles undergo homotypic fusion to form early endosomes (EEs); the EEs accumulate and subsequently form the sorting endosome, where the cargos are selected for recycling, lysosomal degradation or the trans-Golgi network [34]. Depending on the condition, the same receptor often conducts several internalization pathways at once [170,171]. Internalization is an essential process to regulate T cell response, as its deficiency could lead to hyperactivation and rapid exhaustion of T cells [123]. The molecular mechanisms of TCR endocytosis have been under intensive investigation, whereas studies about CARs are emerging.

In the resting state, constitutive internalization and recycling of the TCR complex take place, with CDE as the main mechanism. AP-2 is recruited and binds to AP-2 binding motifs on the ITAMs of CD3 subunits, with higher efficiency to CD3δ [172], or to the di-leucine motif present on the intracellular domain of the CD3γ chain [173,174]. Upon activation, internalization of both antigen-engaged and bystander TCRs is induced. Bystander TCRs convey CDE for internalization and recycling [175], where the cargos are delivered to recycling endosomes for return to the plasma membrane. On the other hand, CIE has been identified as the main pathway of internalization for engaged TCRs, which guides engaged TCRs to late endosomes (LEs), at least partially, for lysosomal degradation [35,176]. Thus, it has been proposed that the balance between recycling and degradation seems to be regulated by the strength of activation [34]. However, which CIE mechanisms are specifically involved in TCR internalization remains unclear. It has been demonstrated that TC21 (Rras2) is associated with TCR and is necessary for TCR internalization from the IS through a RhoG-dependent mechanism [177]. Lysosomal degradation of the receptor is carried out through TCR ubiquitination, which requires two RING finger E3 ubiquitin ligases c-Cbl and Cbl-b, ubiquitous Rab GTPases, and recruitment of endosomal sorting complexes required for transport (ESCRT) [177]. In addition, TC21 and RhoG have also been shown to play a role in TCR trogocytosis [177], a process by which plasma membrane fragments from target cells or APCs are transferred to lymphocytes [178]. Initially, TCR internalization was considered to terminate TCR signaling. However, increasing evidence support the notion that the internalized receptor continues to signal from specialized endosomes and is critical to sustain TCR signaling [179,180] suggesting a dual role of TCR endocytosis [181].

The trafficking of CARs during activation has been investigated by Li et al. on the CD19-BBζ CAR, and it was shown that engagement of tumor antigens induced rapid ubiquitination of CARs, causing CAR downmodulation followed by lysosomal degradation. By mutating all lysine in the cytoplasmic domain of the CAR to arginine, they could successfully repress CAR degradation while enhancing the recycling of internalized CARs to the plasma membrane. This approach has demonstrated both in vitro and in vivo to promote long-term killing capacity and persistence, and to maintain CAR T cells in a less differentiated state with a metabolic profile enriched in oxidative phosphorylation. This finding also underscores the difference between TCRs and CARs in endosomal trafficking and regulation of signaling. A very recent study has shed light on the relevant mechanism, and a correlation between scFv affinity and trogocytosis has been demonstrated, where low-affinity CAR T cells seemed to be more beneficial as compared to high-affinity CAR T cells for prolonged persistence, as antigen transfer by trogocytosis and subsequent fratricide was reduced by low-affinity CAR T cells [37].

## 3. Part II—A Change in Perspective: From “TCR versus CAR” to “TCR and CAR”

While the previous section was focused on side-by-side comparing TCRs and CARs in order to better understand their mechanism of action and to find possible solutions for current challenges and limitations, the following section serves as a change in perspective: from “TCR versus CAR” to “TCR and CAR”. Indeed, during the last five years, more and more studies aimed at combining TCR and CAR technologies, on the one hand, to understand potential interactions and dependencies between TCR and CAR and, on the other hand, as an approach to strengthen the overall anti-cancer T cell response. The latter is based on the hypothesis that in a combinatorial approach, TCR and CAR could counteract their limitations and eventually harmonize through a synergistic interplay.

### 3.1. Combination of an Endogenous TCR and a CAR

The crucial role of the native TCR for CAR T cell signaling and efficacy was already discussed more than ten years ago [66]. More recent studies have shown that knock-out of the endogenous TCR resulted in reduced persistence of CD19-BBζ CAR T cells in vivo, although early CAR T cell response and signaling were not affected by the lack of the native TCR [182]. On a side note, other studies demonstrated that a lack of the endogenous TCR upon CAR knock-in into the TCR locus even resulted in improved functionality [183]. It was hypothesized that stimulation of the endogenous TCR through xenogeneic mouse tissue (displayed by clinical signs of GvHD) or infectious stimuli, might cause physiological activation of the TCR-positive CAR T cell, thereby prolonging survival and tumor control [182]. Instead of removing the native TCR, Cliona M. Rooney’s group aimed for the opposite: actively engaging and activating the native TCR in CAR T cells by using virus-specific T cells as a source for CAR T cell manufacturing [184,185,186,187]. After it was shown that in vitro stimulation of virus-specific TCRs in CAR T cells prolonged survival and was superior compared to polyclonal activation through CD3-specific antibodies [184], this combination of native TCR plus CAR stimulation was soon implemented in several clinical trials (NCT00085930 [188], NCT00840853 [186,189]). Multi-virus-specific CD19-28ζ CAR T cells were shown to rapidly expand in a virus-load-dependent manner, and CAR T cell proliferation was significantly lower in patients without pre-existing EBV, CMV or adenovirus infection [186]. On a side note, this clinical study was successfully realized without prior lymphodepletion, and not even the two HLA-mismatched patients experienced GvHD, which was explained by the strongly reduced TCR repertoire of the infused CAR T cells. The presence of virus infection and stimulation of the native, virus-specific TCR were hence key to therapeutic success, and instead of leaving it to chance, subsequent approaches aimed at controlled, vaccination-induced native TCR activation in CAR T cells [185,186]. It was reported that in vitro vaccination with VZV peptide mix-loaded DCs even helped to partially recover third-generation GD2-28-OX40ζ CAR T cells, which were already exhausted through a previous CAR target antigen encounter [185]. This is particularly surprising given the fact that repetitive signaling via the native TCR or a CAR is generally thought to cause chronic exhaustion, caspase activation and programmed cell death [190]. The most obvious approach to achieve recovery of exhausted CAR T cells would consequently be to reduce signaling, instead of adding a second stimulus such as via the endogenous TCR. In line with that, the FDA-approved Src kinase inhibitor dasatinib was, for instance, successfully applied to enforce a transient rest in CAR signaling, thereby inducing epigenetic reprogramming and restoring functionality of exhausted CAR T cells [191]. Recent work has shown that the revival of exhausted CAR T cells via the endogenous TCR seems to be dependent on several factors:

First, it was observed that the outcome of native TCR activation was dependent on the CAR structure. Native TCR engagement in virus-specific 4-1BB-bearing CAR T cells led to stronger activation marker expression than in CD28 co-stimulated CAR T cells, ultimately causing TCR downregulation and apoptosis [187]. In accordance with this, several studies showed the beneficial effect of stimulating the virus-specific endogenous TCR in CAR T cells containing a CD28 co-stimulatory domain, verified for different scFvs and also for second- and third-generation constructs [185,186,187]. This is especially surprising as CD28 co-stimulated CARs are generally linked to stronger and more rapid effector function, while 4-1BB is thought to promote more durable CAR T cell response and long-term persistence [159,160,192]. Nonetheless, expression of transgenic full-length CD28 was shown to inhibit activation-induced cell death (AICD) through the upregulation of anti-apoptotic proteins and suppression of CD95L [193,194]. This anti-apoptotic effect was also reported for CARs, co-stimulated through CD28 [195].

The second factor, which seems to influence CAR T cell re-activation during concomitant native TCR stimulation, is the level of TCR antigen and the type of stimulus. In two different syngeneic mouse models, it was observed that dual activation of a CAR and endogenous TCR, led to the upregulation of pro-apoptotic and inhibitory receptor genes in CD8^+^ T cells, consequently resulting in exhaustion and strongly reduced tumor clearance. The CD4^+^ subset demonstrated increased expansion, although persistence of CAR T cells was not increased [196]. Before jumping to conclusions regarding the role of the T cell subset, it is important to have a closer look into the type of TCR stimulus. Since this in vivo work was performed using CD19-28ζ CAR T cells with TCR specificity for HY (male minor histocompatibility antigen) or ovalbumin [196], it was hypothesized that the continuous expression of those non-viral TCR antigens might have caused more repetitive stimulation and consequently the observed exhausted phenotype [196,197]. On the contrary, the previously mentioned studies [184,185,186,187] aimed at engaging the native TCR through vaccination, e.g., via virus-peptide-pulsed DCs or pre-existing virus, instead of endogenous antigen. The very latest study in this context demonstrated that also oncolytic viruses can be used for the stimulation of CD8^+^ CAR T cells via the native TCR. CAR T cells loaded with vesicular stomatitis virus or reovirus exhibited enhanced trafficking, infiltration, persistence and functionality in vivo [197]. Additionally, epitope spreading was stated to cause further expansion of the endogenous tumor-specific potential, thereby protecting against re-challenge with CAR target-negative tumor cells. Similar findings were reported in the context of extensive in vivo studies with transgenic mice, expressing HER2-28ζ CAR and gp100 TCR [198]. The latter was specifically activated through gp100-encoding vaccinia virus, thereby significantly increasing the CAR T cell expansion, persistence and the cytotoxic potential against HER2-expressing target cells. Furthermore, long-term surviving mice also developed immunity against tumor antigens other than HER2, which was attributed to epitope spreading and memory formation.

Finally, recent studies indicate that the early-differentiation status during CAR T cell manufacturing might exhibit a determining factor, as native TCR stimulation through pre-existing virus infection has been shown to be advantageous only in virus-specific CAR T cells displaying a central memory but not an effector memory phenotype [186,189]. This is in line with the generally accepted characteristics of central memory T cells, namely the robust proliferative potential and the long-term persistence [199,200,201]. However, as previously described, functional recovery of already exhausted, and consequently terminally differentiated, CAR T cells was also successfully achieved in vitro using peptide mix-loaded DCs [185]. This contradictory result might have again been influenced through the type of TCR stimulus used.

The discussed studies (summarized in Table 2) underline the potential of dual stimulation through CARs and native TCRs, not only raising the question of whether CAR T cells could also be activated through vaccination targeting a transgenic TCR but also, in general, whether there is a beneficial or even synergistic effect of combining a CAR with a tumor-cell-targeting transgenic TCR.

### 3.2. Combination of a Transgenic TCR and a CAR

As reviewed in the previous section, the approach of dual native TCR and CAR stimulation primarily aimed at increasing efficacy and persistence of the CAR T cells, while the paramount objective of combining a transgenic TCR and a CAR has been targeting multiple antigens and hence reducing the risk for immune escape [202,203]. The relevance of this is underlined by the fact that although CD19-targeting CAR T cell therapy has led to extremely promising responses, relapse was observed in half of the patients, primarily due to loss of CD19 surface expression [7]. As hypothesized by Carl June, the likelihood of tumor escape through both downregulation of the MHC and loss of the CAR target antigen is expected to be very low, which ultimately means that in dual-specific T cells at least either one, transgenic TCR or CAR, would still work [204]. In 2016, it was shown for the first time that a CAR and a transgenic TCR can be functionally co-expressed in the very same T cell [202]. The CD8^+^ T cells, which were transiently transfected with a CSPG4-28ζ CAR and a gp100 TCR, led to antigen-specific cytokine secretion and target cell lysis in vitro. The functionality was similar to CAR- or TCR-only T cells, suggesting that co-expression of the CAR and the transgenic TCR did not cause reciprocal inhibition. It is important to highlight that simultaneous stimulation of the CAR and the transgenic TCR resulted in increased cytotoxicity. Interestingly, this effect was shown to be dependent on the co-existence of both receptors on the very same T cells, as this was not achieved with a 1:1 mixture of CAR T cells and TCR T cells (normalized to the total cell count). On the contrary, subsequent work with dual-specific T cells expressing the very same receptors demonstrated reduced cytolytic activity compared to T cells only expressing CSPG4 CAR or gp100 TCR [203]. It is not clear whether this opposed outcome was due to the different types of TCR delivery (lentiviral transduction instead of transient-electroporation-based transfection) or to general variations in the experimental set-up such as the use of another target cell line. However, both studies showed that dual-specific CAR- and transgenic-TCR-expressing T cells specifically recognized and lysed single- and double-positive target cells, representing a potential solution against tumor escape.

Furthermore, several studies demonstrated the beneficial effects of combining a transgenic TCR with a non-classical, modified CAR [205,206,207]. In vivo experiments, for example, displayed that expansion and efficacy of NY-ESO-1 TCR-transduced T cells can be enhanced through co-expression of scFv-lacking 4-1BBζ CAR [205]. In this regard, it was hypothesized that the CAR, albeit not engaging any target antigen, induced a certain trans co-stimulation, thereby enhancing transgenic TCR signaling. Another study demonstrated that the serial killing potential and in vivo anti-tumor response through both native or transgenic TCR was increased by co-expression of CD19 CARs with 4-1BB co-stimulation but lacking the CD3ζ signaling domain [207].

To sum this up, the conclusion can be drawn that a CAR and a transgenic TCR cannot only be functionally co-expressed in the very same T cell, thereby enabling dual specificity and counteracting tumor escape, but might also synergize in a mutually reinforcing way. To this end, further studies evaluating the combination of a transgenic TCR and a CAR (summarized in Table 3) will be crucial to deepen the understanding in regard to the influence on signaling, activation, exhaustion and persistence. While therapy-associated toxicities such as cytokine release syndrome and immune effector-cell-associated neurotoxicity syndrome are major risks in CAR T cell immunotherapy, such strong immune activation is generally not expected during treatment with TCR T cells [208,209]. This explains why additive effects of cytokine-related toxicity are unlikely, although the combination of a transgenic TCR and a CAR results in dual and potentially simultaneous stimulation. Therefore, further studies should rather be focused on exhaustion and persistence, instead of abnormal immune activation. However, clinical guidelines for the management and prevention of cytokine storms are well established [210]. Off-target effects due to the mispairing of transgenic and endogenous TCR chains and the potential risk for uncontrolled formation of new specificities were described as safety problems during TCR gene therapy [211]. Knock-out or replacement of the endogenous TCR by the transgenic TCR represent possible solutions, which might also be applied in a combinatorial approach with CARs and transgenic TCRs [183,212,213].

## 4. Conclusions

Adoptive T cell therapies represent a powerful tool in modern medicine with a so far unrivaled potential to cure patients suffering from advanced hematological malignancies and solid cancer as shown in several clinical trials (CAR T cell trials NCT02228096, NCT02445248, NCT02348216, NCT03391466; TCR T cell trials NCT03159585, NCT02280811, NCT02992743). Interestingly, in the context of this clinical success, a clear trend emerged quite early demonstrating that inherent features of TILs and TCR-engineered T cells resulted in durable and complete responses when treating solid cancer [11,15,214,215,216], while, for instance, the “poster child for CAR therapies” [217], the CD19-directed CAR, led to complete remissions of >88% when treating leukemia and lymphoma in clinical trials. Therefore, aiming to better understand this discrepancy among both technologies, we specifically discussed the current knowledge about TCR and CAR signaling and encouraged a change in perspective from “TCR versus CAR” to “TCR and CAR” as a next step to further ameliorate the potential of genetically modified T cell therapies by counteracting the limitations of CARs and TCRs, respectively. The generation of such T cell products, however, still requires a deeper understanding of T cell signaling and T cell biology, especially in the context of genetically engineered T cells. Accordingly, further dedicated studies including advanced multi-omics analyses are indispensable to further evolve adoptive T cell therapies toward successful treatment of a broad range of cancers.

## Figures and Tables

**Table 1:** Summary of similarities and differences between TCR and CAR.

		TCR	CAR
**Structure**	Receptor clustering	One pMHC potentially enough [18,19]	Clustering required [20,21,22]
	ITAM number	10 ITAMs provided by the CD3 complex [23]	Up to 3 ITAMs per CAR [23]
	Affinity/ Sensitivity	Lower affinity, higher sensitivity	Higher affinity [24], lower sensitivity [25]
	Phosph. of CD3 subunits	Phosph. of CD3 ζ, γ, δ, ε [26]	Phosph. of only CD3 ζ [25]
**Signaling**	Phosph. of signaling molecules	Stronger phosph. of ZAP-70, ITAMs and PLCγ1 than in CAR [27,28]	Stronger phosph. of Lck and ERK than in TCR [29]
	Recruitment of signaling molecules	More efficient recruitment of ZAP-70, CD2 and LFA-1 than in CAR [27,28]	Less dependent on LFA-1:ICAM-1 interaction and LAT [21,25,29]
	Upon increased antigen exposure	Maintain an earlier differentiation phenotype upon strong stimulation [30]	Higher levels of co-inhibitory molecules upon activation [30]
	IS structure	Classical “bull’s eye” structure [29] or multifocal structures formed by Th2 cells [31] or at the interface with DCs [32]	Non-classical, disorganized IS with multifocal pattern [29]
**Immunological Synapse**	SMACs	Conventional IS consisting of cSMAC, pSMAC and dSMAC [33]	Merged cSMAC and pSMAC, no adhesion molecule ring [21,29]
	Lck	One central Lck cluster [29]	Disorganized Lck patches [29]
	Duration	Usually slower/weaker effector function [29,30]; longer IS duration, slower off-rate from target [29]	Faster cytotoxic granule secretion and faster resolution of IS [29]
	Resting state	Constitutive internalization of TCR complex through clathrin-dependent endocytosis (CDE) [34]	Unknown
**Trafficking**	Upon activation	Engaged TCRs: Clathrin- independent endocytosis (CIE) for internalization, recycling or lysosomal degradation [34,35] Bystander TCRs: CDE for internalization and recycling [34,35]	Engagement of antigens induced rapid lysosomal ubiquitination [36] High-affinity CAR T cells demonstrated enhanced trogocytosis [37]

**Table 2:** Overview of studies combining endogenous TCR and CAR.

Combination of Endogenous TCR and CAR					
CAR	TCR Specificity	TCR Stimulus	Study	Main Result	Citation
GD2-ζ	EBV- specific TCR	Patients with EBV pre-infection	NCT000 85930	Prolonged survival and expansion compared to anti-CD3 antibody activation	Pule et al., 2008 [184] Louis et al., 2011 [188]
CD19-28ζ	EBV- specific TCR	EBV-transformed lymphoblastoid B cell lines	NCT008 40853	Stimulation of native TCR increased CAR T cell expansion; T cells were donor-derived after allogeneic HSCT (no GVHD)	Cruz et al., 2013 [189]
GD2-28-OX40ζ	VZV- specific TCR	VZV peptide mix-loaded DCs	In vitro	Exhausted and dysfunctional CAR T cells recovered upon stimulation of native TCR	Tanaka et al., 2017 [185]
CD19-28ζ	HY- specific TCR	Male bone-marrow-derived cells (HY)	In vivo	Dual stimulation led to exhaustion and apoptosis in CD8^+^ (not in CD4^+^) CAR T cells	Yang et al., 2017 [196]
Her2-28ζ	gp100- specific TCR	Recombinant vaccinia virus encoding gp100	In vivo	Increased expansion, persistence, tumor infiltration and functionality upon native TCR stimulation	Slaney et al., 2017 [198]
(1) GD2-ζ (2) GD2-28ζ (3) GD2-BBζ	VZV-/EBV- specific TCR	VZV or EBV peptide mix-loaded DCs	In vitro	TCR stimulation led to increased expansion and functionality in GD2-28ζ (but not in GD2-BBζ) CAR T cells	Omer et al., 2018 [187]
CD19-28ζ	EBV- specific TCR	Patients with EBV pre-infection	NCT008 40853	Virus load-dependent increase in CAR T cell expansion	Lapteva et al., 2019 [186]
EGFRvIII-28-BBζ	Oncolytic VSV or reovirus	Oncolytic virus co-administered with CAR T cell	In vivo	Enhanced trafficking, infiltration and functionality; long-term effects through in vivo reactivation with TCR-directed oncolytic virus	Evgin et al., 2022 [197]

**Table 3:** Overview of studies combining transgenic TCR and CAR.

Combination of Transgenic TCR and CAR					
CAR	TCR Specificity	CAR/TCR Stimulus	Study	Main Result	Citation
CSPG4-28ζ (transient)	gp100 (transient)	Target cell line	In vitro	Functionally co- expressed, without reciprocal inhibition	Uslu et al., 2016 [202]
CSPG4-28ζ (transient)	gp100 (stable)	Target cell line	In vitro	Functionally co- expressed; reduced cytotoxicity compared to TCR T cells	Simon et al., 2019 [203]
BBζ (lacking scFv)	NY-ESO-1	TCR-target- expressing cell line	In vitro/ in vivo	Increased proliferation and tumor regression upon single and repeated TCR stimulation	Miyao et al., 2018 [205]
(1) CD19-28 (2) CD19-BB (3) CD19-28-OX40 (lacking signaling domain)	Survivin	Target cell line	In vitro/ in vivo	Enhanced apoptosis with CD19-BB CAR; CD19-28-OX40 (not CD19-28) increased repeated killing and prolonged tumor control in vivo	Omer et al., 2022 [206]
